# Thermography based skin allergic reaction recognition by convolutional neural networks

**DOI:** 10.1038/s41598-022-06460-9

**Published:** 2022-02-16

**Authors:** Łukasz Neumann, Robert Nowak, Jacek Stępień, Ewelina Chmielewska, Patryk Pankiewicz, Radosław Solan, Karina Jahnz-Różyk

**Affiliations:** 1 Institute of Computer Science, Warsaw University of Technology, ul. Nowowiejska 15/19, 00-665 Warsaw, Poland; 2 Milton Essex S.A., ul. J. P. Woronicza 31/348, 02-640 Warsaw, Poland; 3 Military Institute of Medicine, ul. Szaserów 128, 04-141 Warsaw, Poland

**Keywords:** Computer science, Software, Skin manifestations, Medical imaging

## Abstract

In this work we present an automated approach to allergy recognition based on neural networks. Allergic reaction classification is an important task in modern medicine. Currently it is done by humans, which has obvious drawbacks, such as subjectivity in the process. We propose an automated method to classify prick allergic reactions using correlated visible-spectrum and thermal images of a patient’s forearm. We test our model on a real-life dataset of 100 patients (1584 separate allergen injections). Our solution yields good results—0.98 ROC AUC; 0.97 AP; 93.6% accuracy. Additionally, we present a method to segment separate allergen injection areas from the image of the patient’s forearm (multiple injections per forearm). The proposed approach can possibly reduce the time of an examination, while taking into consideration more information than possible by human staff.

## Introduction

According to the World Health Organization (WHO), allergy is the third most common disease, it is classified as a threat to civilization. The twenty-first century is called the age of allergy epidemics. Experts at the European Academy of Allergy and Clinical Immunology predict that by 2025 over 50% of the European population will suffer from various types of allergies^1^. Unfortunately, more than half of allergy sufferers lack proper diagnosis and, consequently, also treatment^2^. In this context, a significant problem is the insufficient number of allergy specialists, for which effective compensation is possible by increasing the degree of automation of allergy diagnosis as well as by transferring tests from specialized centers to doctors and primary care centers.

Along with medical history, allergy tests may be able to confirm whether a particular substance is causing symptoms. Currently in clinical practice the widely used in vitro laboratory test allows indirect diagnostics of only one type of allergy by determining the level of specific IgE antibodies in human serum or plasma involved in the type I (immediate) allergic reaction^3^. The second technique is an in vivo functional method in the form of skin tests, allowing for the diagnosis of both type I (skin prick test—SPT) and type IV (delayed - patch test) allergies^4^. In this method tiny amounts of allergen are dropped into a small puncture made by a lancet. The area around the puncture is then observed for signs of an allergic reaction. The wheal and erythema regions are marked and measured using a ruler and related by trained professionals to the size of the histamine control and reported on a 0-5 scale. Because skin tests of various types allow mapping the actual response of the patient’s immune system to the allergen it is assumed that they are a clinically reliable predictor of individual hypersensitivity to the tested substances^5^.

Unfortunately, both methods have limitations, can lead to wrong diagnoses, are invasive and require trained professionals to perform the test and to interpret results.

The skin test results are observed by the doctor and, based on a subjective visual assessment of the symptoms, associated with an allergic skin reaction. This applies the difficulty of establishing a standardized measure for the observed key skin allergic reaction in the form of the so-called allergic bubble. The bubble size depends on the technique of puncturing the patient’s skin during allergen application^6^. Moreover, a bubble is formed under the influence of fluid penetration from the skin’s microvessels dilated by the released histamine, but it may also appear in other situations such as toxic dermatitis or even at the site of a negative test; a bubble is non-specific symptom.

In the last decade, new methods have been intensively sought to produce readings of skin allergy tests, using reliable markers that ensure repeatability of the results. There is significant potential for non-invasive far infrared imaging (LWIR), for which relatively shallowly located allergic skin reactions are completely transparent and can easily be recorded with appropriately sensitive instruments^7^. Infrared imaging has been helpful in non-invasive diagnosis of breast cancer, melanoma skin cancer, and skin burns^8^.

LWIR skin imaging enables recording the hyperthermic allergic reaction accompanying the posthistamine perfusion effect)^9^. The sensitivity, specificity and accuracy of thermovision tests were assessed as high^10^, with very high agreement between the reference method used in the clinic and in vitro sIgE results.

Our solution uses a visible and an infrared camera simultaneously, therefore allergic bubble and an skin allergic reaction could be both observed. The data are collected using a new device, where visible and thermal pictures are calibrated at pixel level. The analysis uses a series of pictures taken both before and after applying the allergens. Moreover, the general medical investigation data, like age, gender, body temperature, are used.

The system works on the images of the entire forearm, with multiple allergens per forearm. The algorithms generate fragments of image for single application, called segments, and classify each of them separately. The segmentation process is based on U-Net architecture, while classification model is a custom convolutional architecture.

To the best of our knowledge, there are not any comparable state-of-the-art solutions. Some artificial intelligence models supporting allergists are available. For example, in^11^ neural network supports the diagnosis of the $\beta$-Lactam Allergy. Visible-spectrum images were classified by neural network models to detect more widespread skin diseases^12,13^. There were attempts to correlate thermal and allergic reactions^10,14,15^, but they were purely exploratory and did not introduce an end-to-end automatic approach to recognizing allergic reactions. One study that attempted to classify reactions used the patch test approach in which allergen-soaked pads were put on the patient’s back, and the underlying skin was analyzed using FLIR ONE application^16^. Notably, both studies were done on datasets substantially smaller than ours.

The contribution of this paper can be summarized in the following points:

(I) Usage of thermographic images to the recognition of allergies in humans;
(II) Novel, automatic, and objective method to mark allergies in prick tests;
(III) Comparison of different light spectras (thermal and visible) as an input to the neural network classifier.

We show that using the proposed method we’ve obtained nearly perfect results in terms of AUC (0.98) and AP (0.97). As previously stated, there are not any state-of-the-art solutions. One similar research tested patch-based approach and reported significantly worse results (AUC 0.85). However, it is unclear if these methods can be directly compared, as they differ in the way allergen is introduced to the patient’s body.

This article is organized as follows. “Input” section depicts the input to the system. “Segmentation method” section depicts the segmentation algorithm, “Classification methods” section provides the neural network description used for classification, as well as training and validating techniques. “Results” section describes the dataset of thermal and visual images with full medical documentation, as well as the results. Finally, the discussion is provided in “Discussion” section.

## Input

The system takes three main components as an input, namely a pair of thermographic images, a pair of visible-spectrum images and tabular data. All of them show the patient’s forearms at two moments in time, called a series, before allergen application and 15 min after allergen application.

**Figure 1:**
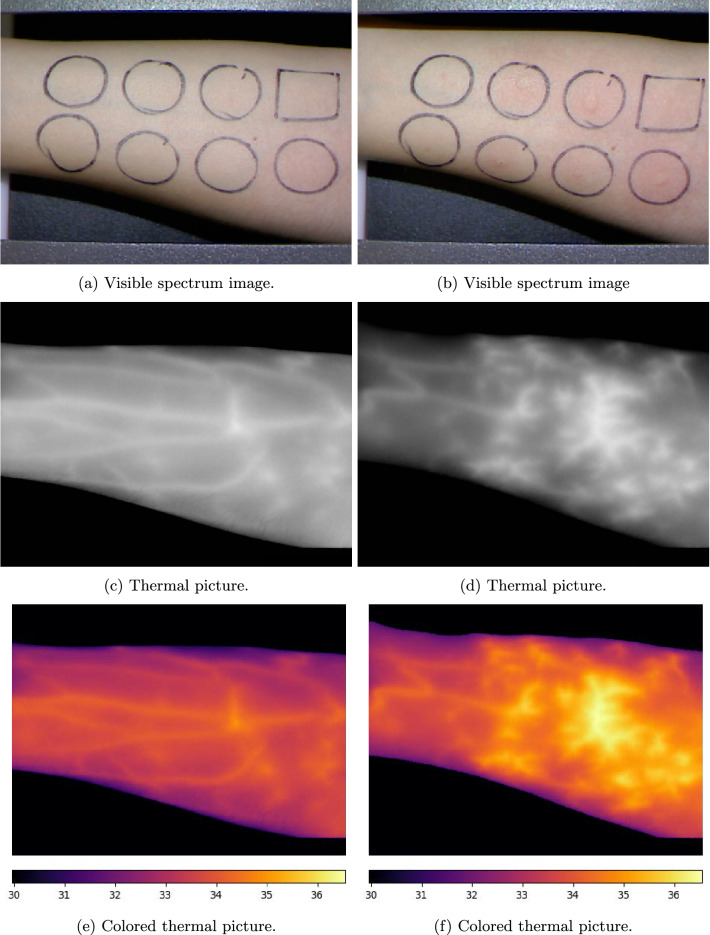
Photos of patient’s forearm before (**a**, **c**, **e**) and after (**b**, **d**, **f**) allergen application. Colorbars in (**e**, **f**) show the temperature scale in Celsius degrees.

Visible-spectrum images and thermographic ones, which are taken at the same time, are correlated on the pixel level. There is not such correlation between series. Device used to produce these images is described in “Dataset” section. Samples of the images are shown in Fig. 1.

Thermographic photos have a single channel. Each pixel corresponds to a temperature reading from the thermographic camera. Visible spectrum images focus on a patient’s skin and hives which in some cases appear as result of allergen injections. The second use of these images is to localize areas where allergens have been injected, using marks drawn on the arm by the person performing the examination.

The data describes patient’s sex, age, weight, height and body temperature measured at the beginning of an examination.

## Segmentation method

### Segmentation problem overview

The purpose of the segmentation process is to find the general areas in which allergens were applied, because the dataset does not contain such information. It’s necessary to find or approximate the positions of all allergens on the forearm, because the ground truth is directly tied to each allergen separately and the classifier evaluates each allergen separately. The positions of the allergens are marked on the skin on the patient forearms’ as squares (histamine) and circles (other allergens and negative control) by a nurse before the examination. As patients’ forearms differ in shape it is not possible to use a fixed grid pattern to find allergen positions. Moreover, a patient has the ability to slightly change the angle of the forearm once it is fixed in the machine, as well as move it forwards/backwards (that is along the horizontal axis on the images). This is depicted in Fig. 1a,b.

The main segmentation problems are hairy forearms, incomplete marking for application positions, and different marker colors. Due to these problems classic segmentation methods, such as color-based segmentation or Canny edge detection^17^, do not yield satisfactory results.

### U-Nets

**Figure 2:**
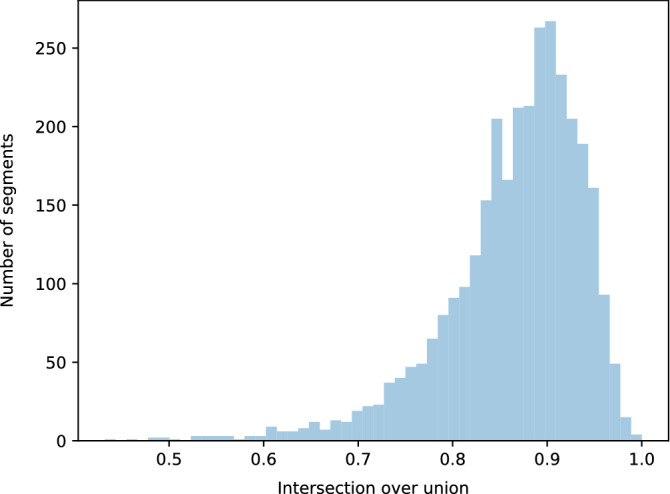
Normalized intersection area between manual and predicted segments.

We use the U-Net^18^ model as a basis for the segmentation process. We used the original architecture proposed by the authors. We manually mark all the allergen application areas by saving the injection point and a point on the highlighter marker. Based on this information we create binary masks, where each application area is marked by a circle. This is a simplification, as the real shapes of highlighter markers differ slightly (most notably rectangle for the histamine), however the resulting model yields acceptable results, as depicted in Fig. 2.

We use the ADAM^19^ optimizer with binary cross-entropy (BCE) loss. The model is trained on images downscaled to $512\times 385$ (that is, halved in size) with pixel values normalized to the range [0, 1]. We train the model for 30 epochs.

Images that are the result of U-Net segmentation are then fed to the contour-searching algorithm^20^. Next, we filter out contours with appropriate size and shape (based on the bounding box).

Finally, in cases where not all markers are found, we use a heuristic approach to approximate the allergen grid based on the segments found and fill in the missing ones. The algorithm chooses segments using contours and bounding rectangles with appropriate ratio and size. Next, segments are arranged in the grid by splitting them into two lines and finding proper positions by taking the offset from the right side. After that, the process searches for a minimal length vector between the positioned segments and fills in the missing ones, adding such vectors to the center of neighboring segments.

## Classification methods

### Delta thermal images

Thermal images tend to contain features that can be indistinguishable from allergic reaction (e.g. blood vessels). We call image created by subtracting pre-reaction image from the post-reaction one a ‘delta’ thermal image. Ideally such image should contain only thermal changes caused by the allergic reaction, assuming that patient’s environment does not change. To create delta image pre- and post-reaction images need to be aligned. This is because it is not possible to take an image of a patient’s forearm in exactly the same position two separate times. Patients have some leeway to change the angle of the forearm, they can also rotate it slightly, as well as position it differently with respect to the arm restraints placed near the elbow and wrist of the forearm. This effect can be observed in Fig. 1a,b.

We tested two approaches to image alignment. In both techniques we exploit the fact that after the segmentation phase we have eight separate markers per image. The basic premise is to find a transform which would align two sets of markers between pre-reaction and post-reaction images. Note that this requires sensible segmentation results - in order for this approach to work we need to ensure that enough markers are found on both images and that these are matching markers. The more markers are found the better the alignment results.

In the first technique a homography matrix is estimated based on the sets of markers.

The second approach is simpler in terms of degrees of freedom. First, linear regression is fitted on each set of markers. This gives us two lines, each for pre- and post-reaction images. Next, a transformation consisting of translations and rotations is calculated. This transformation should minimize the distance between two lines.

Regardless of the technique used, once the transformation matrix is estimated, it is used to transform the pre-reaction image. Finally, a delta thermal image is created by means of subtraction.

### Classifier input, preprocessing and augmentation

The classification model works on a single segment images, that is for each found segment (allergen) on an image we cut out a separate region of interest and save it. We use segments found on the visible-spectrum images to cut out matching segments on thermal images, under the assumption that corresponding visible-spectrum and thermal images are pixel-perfectly correlated. For each segment found, the region of interest (ROI) is a $300\times 300$ square, with the center taken from the center of the segment. An average size of the bounding box for the segment is 85 pixels in our dataset ($\sigma = 6$, min 60, max 104), so ROI includes a substantial padding. This helps us to account for the minor inaccuracies in the segmentation method, as well as add context to information about the surroundings for the network. See “Discussion” section for more information about why this is important.

A sample in the dataset for the classification task consists of patient’s attributes and two $300\times 300$ images—a visible-spectrum one and a corresponding delta image. Attributes used for the classification are recorded from an interview—sex, age, weight and body temperature. Note that all samples for a single patient will share the values of these attributes, as they are not allergen specific. The ground truth for each sample is a binary label created based on the diagnosis of doctor-allergologist.

Patients’ attributes are rescaled to the range [0, 1]. Images are normalized with respect to the mean and standard deviation. Statistics used to normalize and rescale data are calculated on the training dataset and used for the test dataset.

The following augmentation methods are used for the images: random horizontal and vertical flips, random rotation for up to 45 degrees, and random translation up to 4 pixels, random zoom up to 1.3 ratio. Each transformation has a 50% chance of being used with parameters for specific transformation drawn from uniform distribution. Notably, if the model is trained on both visible-spectrum and thermal images then images for each sample are augmented identically (i.e. each sample has two images and both of them will be augmented in the same way, both in terms of which augmentation methods are used and specific parameters for these transformations).

### Neural classifier

Classification is based on a convolutional neural network. As described in “Classifier input, preprocessing and augmentation” section a single sample consists of two images—one with three channels and one single-channeled, together with a patient’s attributes. In our experiments we test using either one of the images, or both. In the latter case (both images used) we fuse images together after preprocessing and augmentation steps into a single four-channel tensor. The patient’s attributes are fed into the first fully-connected layer.

The proposed architecture of the convolutional subnetwork is depicted in Table 1. We use average pooling layers with $3\times 3$ kernel and stride 2. All convolutional layers use $3\times 3$ kernels and are followed by batch normalization. The number of input channels is changed on the basis of the input data—four channels for both images used, one channel for thermal-only classification and three channels for visible-spectrum-only one. The fully connected subnetwork comprises of a Dense layer with 64 output channels followed by a LeakyReLU, Dropout and Dense layer with a single output channel.

We use Leaky-ReLU activation functions for all convolutional and dense layers except for the output layer, which uses softmax activation. ADAM optimizer with decoupled weight decay regularization^21^ is used. Weight decay is set to $10^{-4}$, while the negative slope of Leaky-ReLU is $10^{-2}$ and the learning rate is set to 0.001. The dropout value between two dense layers is 0.5. Cross-entropy loss is used as a loss function.

Training is early-stopped after 80 epochs, as the model has a tendency to overfit, as seen on Fig. 4.

**Table 1:** Output channels for the convolutional part of the network.

Output channels	Pooling after convolution
32	Yes
64	Yes
64	
128	
128	Yes
256	
256	Yes
256	
256	Yes
256	
256	Yes

All layers (including pooling) have $3\times 3$ kernels. All convolutional layers are followed with batch normalization and LeakyReLU activation.

The proposed architecture was selected as the best performing from among several state-of-the-art architectures: MobileNetV2^22^, DenseNet-BC-76, DenseNet-BC-121^23^, ResNet-18^24^, where each architecture was used without the original fully-connected layer(s). Instead their convolutional parts were used, with four-channel inputs. All models used the same fully-connected layers, as proposed in our architecture.

### Ethics

The data collection procedure was approved by Komisja Bioetyki przy Wojskowym Instytucie Medycznym, protocol number 15/WIM/2017 on the 15.03.2017. The procedure was performed with accordance to the guidelines and regulations of the said commision. Informed consent was obtained from all participants.

## Results

### Dataset

The data were collected throughout years 2018–19 in the Clinic of Internal Medicine, Pneumonology, Allergology and Clinical Immunology of the Military Institute of Medicine, Warsaw, Poland. To illustrate allergic hyperthermia, the InfraScan$^{\mathrm{TM}}$ instrument developed by Milton Essex SA was used. Milton Essex SA is a company partially funding this research. InfraScan$^{\mathrm{TM}}$ has a high-resolution thermovision measuring system with an uncooled microbolometric matrix, enabling the recording of hyperthermic skin reactions with a diameter of less than 0.3mm. The instrument was developed for capturing allergic reactions, technical data of the thermovision system can be found in Supplementary Tables S1 and S2 online.

This device consists of a visible-light camera correlated with thermodetectors at pixel level and a frame with restraints near the patient’s wrist and elbow. The restraints help immobilize the forearm and allow for smaller variations in the position/tilt of the forearm on the images. Despite these measures, patients still can slightly rotate the forearm and medical staff needs to make sure that restraints are put in similar places each time the image is taken.

To gather the images, first, the allergen application fields were marked on each of patient’s forearms. Next, images (both thermal and visible-spectrum) of the patient’s forearms were taken before the allergen application. The allergens were then applied, and the patient was asked to wait for 15 min in a sitting position. After this rest period, the images of both forearms were again taken. To account for various environmental factors the following steps were followed throughout the data gathering process:

1. Prior to the examination the device was placed in the examination room for 60 min to stabilize the temperature of its cover and detection hardware.
2. The examination was performed in a closed, air conditioned room without intensive air flow. The temperature and humidity in the room were stabilized and measured respectively ${22}\;^{\circ }{\mathrm{C}}$ and $45\%$. Specialized air conditioning and humidifier systems were used to ensure stable conditions throughout the examination process.
3. Acquisition of images in each sequence was preceded by an automatic, single-point recalibration procedure. This procedure uses a blackbody temperature reference placed inside the head of the device. Additionally, it also corrects thermal drift based on data from sensors placed around microbolometric matrix and proprietary algorithms.
4. Patients were qualified for the examination based on the various medical premises. Additionally, all physiological aspects of the thermoregulation process were taken into account. These included drugs and other substances influencing body temperature and thermogenesis. Before the examination, each patient spent 15 min in a waiting room with a specialized air-conditioning humidification system with a temperature and humidity precisely like the temperature and humidity in the examination room. During the examination, the patient was in the examination room.

The details of the data gathering process were described in the study protocol MESX/ISC/09/18 (Infrared Imaging of Field of Allergic Reaction).

The dataset consists of interviews and photographs gathered from 100 participants (47 men and 53 women), who underwent a series of skin prick allergy tests with 12 allergens. In total 404 thermal and 404 visible spectrum images were gathered. All photos are sized $1024\times 770$ pixels. Samples were split equally between two regions: right and left hand. For control purposes, a histamine (positive sample) and negative control sample were used along with allergens. In total eight allergen application fields were placed on the patient’s forearm, which resulted in 32 samples from all regions and series. The whole dataset contains 1584 allergic reaction samples with 501 (31.6%) positive and 1083 (68.4%) negative cases. A total of 1600 segments were obtained, including hyperthermic allergic reactions as well as histamine and negative control, however some were discarded due to technical issues in the questionnaire.

The last part of the dataset is a collection of interviews. It contains basic information about each patient and diagnoses of allergic reactions. All the substances used, along with histamine and negative control samples, are listed in an interview. The region they are associated with and their position on the patient’s forearm, which are part of the data collected, enable the matching of the doctor’s diagnosis to fragments of images corresponding to proper reactions. This diagnosis is used to create binary ground-truth labels for the classification task.

To obtain segmentations for the entire dataset we run a leave-one-out cross-validation on the U-Net model and save the calculated segments.

### Evaluation methods

We use Intersection over Union (IoU) to estimate segmentation results. For each segment we calculate the intersection area between a manual segmentation and an automatic one, as well as area of union. IoU is then calculated as $\frac{intersection}{union}$.

To evaluate our classification approach we use ten-fold cross-validation as well as leave-one-out cross-validation (LOOCV). To avoid a situation in which the model has seen other parts of the hand in the test set we can not simply stratify and fold over reactions. Instead we fold over patients, ensuring that the model has not seen either other parts of the hand or the patient’s attributes. The mean and standard deviation used to normalize data are calculated on the training part of the data for each split. A model evaluation is based on the receiver operating characteristic (ROC) and precision-recall curve (PRC) and their corresponding numerical statistics—area under the curve (AUC) for ROC and average precision (AP) for PRC. Additionally, we check the accuracy of the model on a threshold that is selected to maximize the F1 score (in practice close to 0.5).

### Segmentation

**Figure 3:**
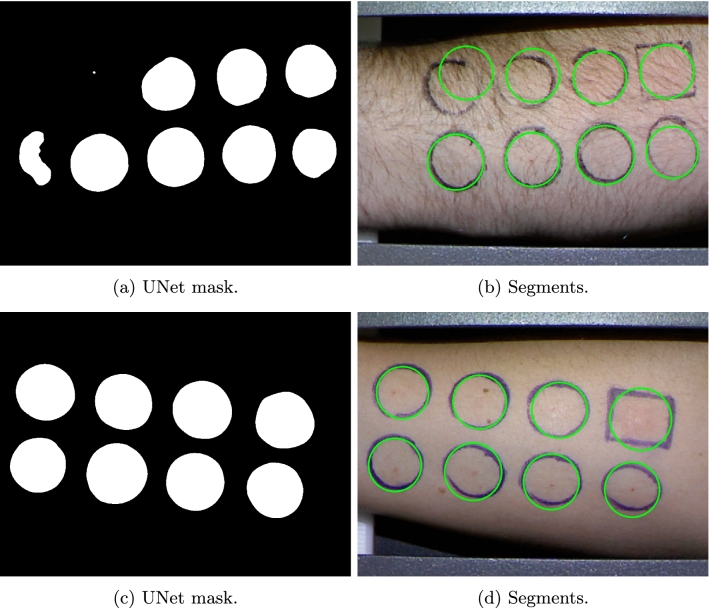
Sample segmentation results for problematic image. First row shows the result for a hairy forearm, while the second row shows the result for a different marker color (blue).

Results of segmentation are satisfactory, as presented in Figs. 2 and 3. In practice we do not need to achieve IoU of 1, as the manual segmentation is always a perfect circle, and due to scaling and arm position it will not always be the case. This fact partially explains why the IoU distribution is centered around 0.9. Moreover, as described in “Classifier input, preprocessing and augmentation” section we add a substantial margin around each segment found and so small deviations from the actual center of the segment should not influence the classification process too strongly.

### Classification

Overall we achieve roughly 93.5% accuracy on the dataset. Detailed results are depicted in Table 2. Notably, the ten-fold validation results are close to the leave-one-out validation results and so we report them for different experiments, as it takes considerably less time to calculate ten-fold validation results. Results indicate that U-Net based segmentation is comparable to manual segmentation. Interestingly, if the model is trained only on thermal images it performs as well as the one trained on both visible-spectrum and thermal images. A model that is trained on only visible-spectrum images performs worse (89.71% vs 93.24%) than the one trained on both types of images.

**Table 2:** Validation results for different types of segmentation and input data.

Input spectra		Segmentation	Validation	ROC AUC	PRC AP	Accuracy (%)
Visible	Thermal					
✓	✓	U-Net	Leave-one-out	0.975	0.956	93.50
✓	✓	manual	10-fold	0.970	0.952	92.79
✓	✓	U-Net	10-fold	0.978	0.961	93.24
✓	✗	U-Net	10-fold	0.940	0.880	89.71
✗	✓	U-Net	10-fold	**0.982**	**0.967**	**93.56**

The best values are in bold.

**Figure 4:**
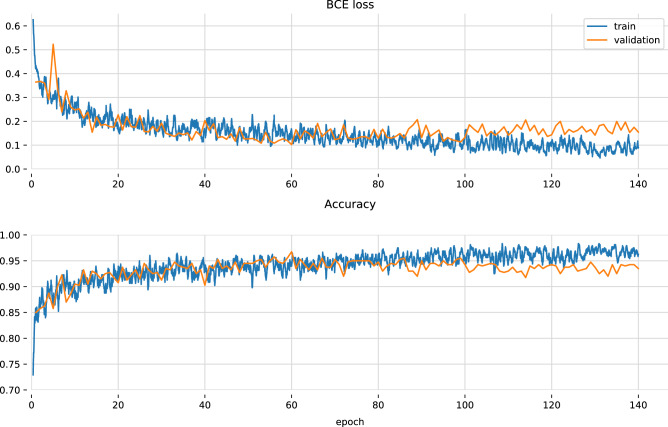
Loss and accuracy of the proposed model based on a single split in the cross-validation procedure.

Loss and accuracy changes throughout the training process are shown in Fig. 4. They were calculated on a single validation split. Since the model has a slight tendency to overfit after approx. 100 epochs we use early stopping. Notably the exact moment of stopping is not as critical since the effect is gradual and there is a span of several dozen epochs during which the training process can be halted.

**Figure 5:**
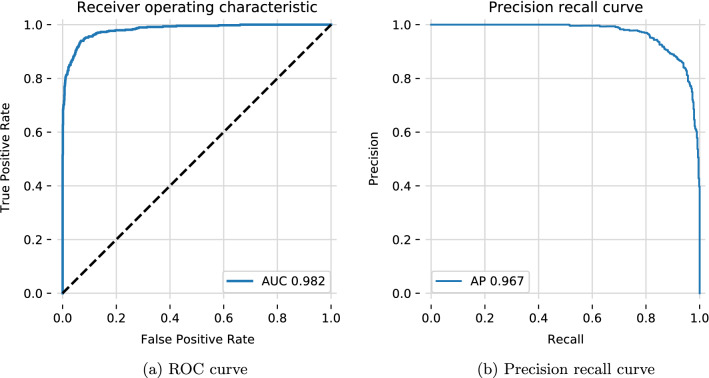
Both figures calculated on the results of the cross-validation procedure for the proposed model, which was built only on thermal images (last row in Table 2).

Precision recall curve and ROC curve are depicted in Fig. 5, while a confusion matrix for the thermal-only cross-validation results is presented in Table 3.

**Table 3:** Confusion matrix for the ten-fold cross-validation, classifier built only on thermal images (last row in Table 2).

True diagnosis	Predicted diagnosis		Total
	Negative	Positive	
Negative	1042	61	1103
Positive	41	440	481
**Total**	1083	501	1584

## Discussion

The results are promising in that we are able to achieve the same model performance both using only thermal images and thermal combined with visible spectrum images. Moreover, the model trained only on visible spectrum images yielded worse results. This indicates that thermographic photography can be used to classify allergic reactions. Additionally, this approach should work in the same way regardless of the skin color of the patient (assuming correct segmentation). One limitation of exclusively using thermal images is that the patient should have a normal heart and respiration rate, as high-intensity physical activity results in images similar to acute allergy reactions.

There are two main problems that influence the results of classification. Hair on the forearm is the first one, as it makes segmentation harder but notably more hair has distinctly lower temperature. This in turn means that, with thick enough hair, the image is somewhat obfuscated and it is harder to recognize allergic reaction patterns. The second problem stems from the fact that blood flows freely throughout the entire forearm, and so it happens that an allergic reaction does not necessarily occur in the place of the injection. This effect can be seen in Fig. 1f, where it is not clear which allergen caused which micro-thermal surge in temperature.

It is possible that with a bigger dataset we could reduce the impact of these factors. One approach that we want to examine in the future is a model, which takes the image of the entire forearm as an input and predicts reaction for each allergen. Currently training such models is not feasible, as the dataset is too small in comparison to the size of a single image which should be used for such an experiment. Using entire-forearm images should help us with the problem of blood flow. Another idea is to align images of the segments with respect to the blood flow. We experimented with that technique, but found that the results were worse, most probably due to the limited augmentation scope. If we align the images then random flips are no longer used and rotations are much smaller (between $[-10^\circ , 10^\circ ]$). With such augmentations and the size of our current dataset model tended to aggressively overfit around the 20th epoch and achieved overall worse results. We want to note that with a bigger dataset this idea could actually boost the results.

In the future we also want to focus on researching the exact time that post-injection images are taken. The current allergological standard is the examination of patients after roughly 15 min post-injection. Our initial research shows that while this is the time required for the on-skin effects to show (i.e. hives and color change), it is not the case for the thermal reaction. We believe that it is possible to change the examination time to approximately 5 min, however this claim needs more research to validate it.

Additionally, we plan on assessing the robustness of the model with respect to high-frequency filtering. We theorize that it should be rather robust in this regard, since reactions do not have sharp edges and sudden gradient changes.

## Supplementary Information

Supplementary Information.
