# Supplementary material for: Thermography based skin allergic reaction recognition by convolutional neural networks
Source: Sci Rep. 2022 Feb 16;12:2648. doi: 10.1038/s41598-022-06460-9 (PMC8850609; doi:10.1038/s41598-022-06460-9)
Supplement: Supplementary file 1 — Supplementary Information. [file 41598_2022_6460_MOESM1_ESM.pdf]

Thermography based skin allergic reaction recognition by convolutional neural networks — supplementary tables

Łukasz Neumann*[1], Robert Nowak[1], Jacek Stępień[2], Ewelina Chmielewska[1], Patryk Pankiewicz[1], Radosław Solan[2], and Karina Jahnz-Różyk[3]

[1] *Warsaw University of Technology, Institute of Computer Science, ul. Nowowiejska 15/19, 00-665 Warsaw, Poland*
[2] *Milton Essex S.A., ul. J. P. Woronicza 31/348, 02-640 Warsaw, Poland*
[3] *Military Institute of Medicine, ul. Szaserów 128, 04-141 Warsaw, Poland*

December 2021

Parameter	Value
Spectrum recording range [LWIR]	8 µm to 14 µm
Resolution of the microbolometer array [LYNRED]	640×480 pixel
Pixel size of the microbolometer array	17 µm
Frame rate	30 Hz
Error of absolute skin temperature measurement	$< \pm 0.1$ °C
Repeatability of temperature measurement	$< \pm 0.05$ °C
Resolution of temperature measurement [NETD]	<35 mK
Spatial resolution (Instantaneous Field of View - iFOV) [mm]	<0.3 mm
Automatic temperature drift correction of IRFPA	Yes (hardware & software)
Lens (Germanium)	25 mm, F#1.4

Supplementary Table S1: Technical data of the infrared module (TIR-01A)

Parameter	Value
Black-body emitter dimensions	50 mm × 50 mm
Absolute (working) temperature range	+10 °C to +25 °C
Relative (differential) temperature range	−15 °C to +50 °C
Emissivity (e)	0.97 ± 0.01
Homogeneity of temperature distribution	<0.01 °C or 0.4%ΔT
Resolution	1 mK
Stability	±3 mK
Temperature measurement uncertainty	<20 mK

Supplementary Table S2: Technical data of the calibration black-body module (MBB-1A)
